# Clinical practice teaching system for MNS cardiac rehabilitation: Delphi consensus

**DOI:** 10.1371/journal.pone.0351886

**Published:** 2026-06-25

**Authors:** Caiyun Sun, Xingbin Du, Yanyan Fan, Guiyun Wang

**Affiliations:** 1 School of Nursing, Binzhou Medical University, Yantai, Shandong, China; 2 School of Nursing, Shandong Xiehe University, Jinan, Shandong, China; Marwadi University, INDIA

## Abstract

**Background:**

China faces a growing demand for cardiac rehabilitation professionals that outstrips available medical resources. While nursing master's candidates represent key talent reserves, current higher education lacks standardized training frameworks for cardiac rehabilitation nursing. This study developed a competency-based clinical training system through expert consensus to address this educational gap.

**Methods:**

A modified two-round Delphi process engaged 22 cardiac rehabilitation experts. Initial expert feedback informed questionnaire refinement, followed by consensus-building analysis in the second round.

**Results:**

The Delphi study demonstrated high engagement (100% Round 1; 96% Round 2 response rates) and acceptable expert authority (Cr = 0.83‑0.84). Kendall’s W values ranged from 0.13 to 0.21 (p < 0.05), indicating moderate agreement among experts. The consensus process yielded a structured framework of 5 domains, 29 subdomains, and 85 competency indicators.

**Conclusion:**

The teaching system is scientifically valid, targeted, and practical, providing a comprehensive framework for training master's degree candidates in cardiac rehabilitation nursing.

## Intronduction

Cardiovascular diseases (CVD) represent the leading cause of global mortality and disease burden, with alarmingly high prevalence and mortality rates [1]. The World Health Organization reports that CVD mortality has been the highest across all income levels, particularly in high-income and middle-income countries [2]. China shoulders the heaviest CVD burden, with over 330 million affected individuals according to the National Center for Cardiovascular Diseases [3]. This epidemic necessitates urgent implementation of evidence-based secondary prevention strategies, particularly cardiac rehabilitation (CR)—a multimodal intervention.

As a multimodal secondary prevention strategy encompassing medical evaluation, exercise prescription, and psychosocial support [4], CR demonstrates dual efficacy in both attenuating atherosclerosis progression and enhancing patients’ functional capacity and quality of life [5,6]. Within the multidisciplinary CR team structure, nurses assume pivotal responsibilities spanning exercise prescription implementation, pharmacological guidance, psychological intervention, and health literacy promotion [7]. In many countries, CR programs predominantly involve nurses undertaking multiple roles, including sequencing multidisciplinary services for each patient at different stages of rehabilitation [8]. American Association of Cardiovascular Pulmonary Rehabilitation regards a CR specialist as a senior practice role the statement “Cardiac rehabilitation nursing is not a beginning nursing position” [9]. While the importance of CR nursing is well established, significant gaps exist in specialized training systems, particularly in developing countries.

China initiated the Master of Nursing Specialist (MNS) program in 2010 to cultivate advanced practice nurses [10]. Compared with countries such as Europe that have established postgraduate courses in CR covering different topics [11], in China current MNS curricula predominantly emphasize training in areas like orthopedics and traditional medicine [12,13], showing limited emphasis on CR specialization. Existing pedagogical research narrowly focuses on procedural skill acquisition rather than systematized competency development—a disconnect impeding the cultivation of expert CR nurses capable of leading multidisciplinary teams. Because the attitude of the medical staff toward cardiac rehabilitation directly affects the therapeutic process [14], setting up CR courses and providing sufficient training on cardiovascular prevention and rehabilitation could greatly improve the attitude of health professionals with low job titles.

Competency-based education (CBE) offers a transformative paradigm shift, prioritizing measurable clinical proficiencies over traditional time-bound curricula [15]. Its three-tiered framework (macro-meso-micro) enables systematic cultivation of advanced competencies through iterative clinical practicums and simulation training. Macro Level refers to the broad goals of CR nursing education. These goals are based on the overall needs of healthcare and CVD patients. Meso Level is about how CR nursing education is organized in schools or hospitals. It includes the design of courses and teaching methods. Micro Level focuses on the specific skills and knowledge that CR nursing students need to learn.

This research considered CBE as the theoretical basis through literature review and Delphi expert (a structured communication technique involving iterative expert surveys) consultation for constructing a clinical practice teaching system for Nursing Master’s students in CR, provide a scientific basis for the reform of CR teaching and personnel training, bridging the chasm between theoretical standards and clinical praxis, our model aims to standardize CR nursing education, ultimately enhancing secondary prevention efficacy for CVD patients.

## Methods

### Design

The Delphi method, an iterative and structured multistage approach, is widely used in health and social sciences to achieve expert consensus, especially when empirical evidence is limited [16]. It involves several rounds of anonymous questionnaires, with each round refining responses based on statistical feedback until consensus is reached.

The traditional Delphi starts with open-ended questions to generate initial items, while the modified Delphi pre-selects items for the first round, often requiring involve two or more rounds of questionnaires [17,18]. This method eliminates geographical barriers through online platforms, ensures anonymity (or quasi-anonymity), and is cost-effective, making it suitable for validating questionnaires, identifying research priorities, and developing clinical guidelines [19]. Researchers act as neutral facilitators, providing impartial information and maintaining objectivity throughout the process. The study was carried out according to the Conducting and Reporting of Delphi Studies checklist guidelines (S1 File).

### Selection and composition of the expert’s panel

The expert panel selection is the most critical stage in a Delphi study, We set the following selection criteria when seeking experts for correspondence consultations: (1) Engaged in nursing management, nursing education, or clinical nursing (cardiovascular or CR); (2) bachelor's degree or above; (3) intermediate or higher professional title; (4) more than 5 years of relevant work experience in corresponding fields; (5) is willing and agrees to participate. To broaden and diversify the perspectives obtained, participants from various regions were selected, provided they met the inclusion criteria, a total of 22 experts were included.

All participants included filled out the survey questions during a two-month period (20 August to 17 October 2022). Researchers invited participants via email with a survey introduction. Upon accessing the study, participants reviewed study details and informed consent, which they had to accept before starting the questionnaire. Anonymity and confidentiality were ensured, and participants were notified of their right to withdraw at any time without consequences.

### Literature review

During the preliminary research, guided by competency – based education theory, a comprehensive search was conducted in multiple databases such as PubMed, Web of Science, and CNKI. Supplementary references included books and training outlines from the Chinese Rehabilitation Medical Association. The research focused on Chinese and English literature about master’s – level cardiac rehabilitation nursing education, excluding conference abstracts and reviews. Relevant literature was identified to lay the foundation for developing the program. Based on the literature search, the initial draft of the clinical practice teaching system indicators for nursing master’s students in cardiac rehabilitation was designed. S1 Table (S2 File) shows the search strategy in PubMed, which was adapted for other databases. This study used Delphi survey to achieve consensus among experts on the Clinical Practice Teaching System Indicators for Nursing Master’s Students in the Area of CR, and the study adhered to the recommendations for the Conducting and Reporting of Delphi Studies (CREDES) [20].

### Delphi consulting and data collection

The online questionnaires were created using Microsoft Forms and consisted of three parts. First was the informed consent for expert consultation. Second, the draft framework had 5 first – level items, 29 second – level items, and 53 third – level indicators, with a 5 – point Likert scale (1 = very not important, 5 = very important) for rating indicator importance, and columns for experts to modify, delete, and expand indicators. The third part was the Expert Basic Information Survey Form, covering details like gender, age, and research direction, plus the expert's familiarity and judgment basis. If experts didn't return the questionnaires within a week, reminders were sent via email or phone to complete the study. In the second round, experts were provided with a revised version of the program, incorporating feedback from the first round. For questions where experts indicated ‘Disagree’ or ‘Strongly disagree’, suggestions for improvement were requested. Inclusion criteria of indicators: Screening criteria with importance mean≥4.00 and coefficient of variation<0.25, the research team needs to discuss and decide whether to retain or delete indicator items [21].

### Data analysis

Data entry, sorting, and analysis were performed using Excel 2016 and IBM SPSS 24.0. For statistical descriptions of experts’ general information and questionnaire scores, measurement data were presented as means and standard deviations, while counting data used frequencies and percentages. The expert positive coefficient was reflected by the effective questionnaire recovery rate.The degree of expert authority is represented by the authority coefficient (Cr) and the average of the familiarity coefficient (Cs) and the basis of judgment coefficient (Ca) [22]. The degree of coordination of expert opinions is represented by coefficient of variation and coefficient of Kendall harmony. The degree of concentration of expert opinion is expressed by the importance score. The order chart method was used to determine the weights of each evaluation index. Statistical significance was indicated (*P* < 0.05).

### Ethical considerations

This study was conducted in accordance with the Declaration of Helsinki (as revised in 2013). The research involved human participants (expert panel members) but did not include patients, clinical interventions, or any identifiable sensitive data. According to the policy of the Institutional Review Board of Binzhou Medical University, formal ethics approval is not required for studies that solely collect anonymous expert opinions through surveys without intervention or patient contact. Therefore, this study was exempt from full ethical review. Nevertheless, all participating experts provided electronic informed consent prior to completing the questionnaire. They were fully informed of the study purpose, the voluntary nature of participation, and their right to withdraw at any time without negative consequences. Anonymity and confidentiality were strictly maintained throughout the study.

## Results

### Demographics of the panel experts

The demographic characteristics of the final sample are presented in S2 Table (S3 File). The age of the experts ranged from 32 to 67, with an average age of 44.82 ± 7.974 years. The average working life was 22.68 ± 9.089 years, ranging from 6 to 43 years. In terms of educational background, 45.5% of the experts held an undergraduate degree, 31.8% held a master’s degree, and 22.7% held a doctoral degree. Regarding employment positions, 63.6% of the experts were in clinical nursing, 4.5% were in clinical medicine, 13.6% were in nursing management, and 18.2% were in nursing education.

### Results of the first Delphi survey

This study developed a clinical practice training system through a two-round Delphi method. Following the first round of expert consultation, the research team optimized the framework based on feedback: redundant content was consolidated (e.g., three assessment and training indicators were merged into two core modules: “Exercise Tolerance and Risk Assessment with Rehabilitation Training” and “Specialized Cardiac Rehabilitation Assessment and Training”), non-core items were eliminated (e.g., removing techniques like “Myocarditis Care” while retaining essential requirements such as “Professional Ethics and Legal Compliance”), specialized competencies were expanded (e.g., adding five third-level indicators including “Post-Cardiac Transplantation Care” and “Aortic Dissection Management”), and training complexity was stratified (e.g., splitting “Electrocardiographic Monitoring and Defibrillation Techniques” into basic and advanced modules). The second Delphi round confirmed consistent expert agreement on all items, finalizing a standardized system comprising 5 first-level indicators, 29 second-level indicators, and 85 third-level indicators.. The detailed indicator lists with importance ratings, coefficients of variation, and weights are provided in S3–S7 Tables (see S4 File).

## Discussion

This study identified the key educational priorities for the clinical practice teaching of Master of Nursing Specialist (MNS) students in cardiac rehabilitation (CR). Using competency‑based education (CBE) as the theoretical framework, we employed the Delphi method to construct a clinical practice teaching system that includes teaching objectives, content, methods, evaluation, and rotation duration. As Moll-Khosrawi et al [23] noted, clinical teaching systems should be developed with broad stakeholder participation. Accordingly, our expert panel included clinicians, nurses, and educators who focus on CR training in their daily work. Establishing a teaching system with detailed, operationalized competency indicators is vital for guiding MNS training in CR and equipping students with the comprehensive knowledge and skills needed to enhance clinical practice competencies.In medical education, setting needs‑based, comprehensive, and achievable teaching goals is critical to curriculum success [24]. We defined teaching objectives across three dimensions: knowledge, skills, and professional qualities. Knowledge objectives ensure evidence‑based care. Students must gain an in‑depth understanding of cardiovascular anatomy, pathophysiology of atherosclerosis, and current treatment concepts [25]. At the same time, the development of practical skills is inseparable from a solid theoretical foundation. Rich theoretical knowledge is essential to guide clinical practice [26]. Skill objectives enable students to apply knowledge clinically. Essential skills include implementing exercise programs, providing patient education and counseling, facilitating lifestyle modification, and conducting follow‑up [27]. Quality objectives focus on professional values and ethics, which shape overall competence and attitude. Medical schools should integrate these quality values into all aspects of training, including clinical practice teaching, to maintain educational quality and ultimately improve patient outcomes and public health [28].

The teaching content covers specialized care for CVD patients, emergency techniques, rehabilitation assessment and training, and nutritional guidance. Lack of CR knowledge among clinical staff reduces patient participation rates [29]; therefore, enhanced education on CR indications and appropriate referrals is essential. For example, not all cardiac conditions (e.g., arrhythmias, ablations) qualify for CR. Our curriculum includes not only pathophysiology and risk factors but also long‑term interventions such as nutrition counseling, weight, blood pressure, lipid, diabetes, and psychosocial management, as well as physical activity counseling and exercise training.. Feinberg et al. [30] developed an adapted CR training program for homebound patients and reported significant improvements in clinicians’ CVD and CR knowledge after training. Standardized cardiac care plans with exercise protocols would help clinicians determine how and when to progress patients and how to monitor responses safely. Our phased, progressive teaching content gradually shifts students’ focus from isolated technical skills to whole‑patient care, avoiding repetition and sustaining motivation and a sense of achievement at each stage [30]. Enhanced education for medical providers focused on cardiac rehabilitation indications and appropriate referrals is needed. For example, not all cardiac diseases have an indication for cardiac rehabilitation, such as arrhythmias, ablations, and so forth. This study taught not only about pathophysiology and risk factors for heart disease, but also about interventions and their long-term benefits, such as nutrition counseling, weight management, blood pressure management, lipid management, diabetes management, psychosocial management, physical activity counseling, and exercise training. These interventions are taught at a medical school level and emphasized during primary care curriculum, but the connection to cardiac rehabilitation remains disjointed [31].

Teaching methods should enhance students’ learning experience, interest, and engagement, thereby improving teaching effectiveness [32]. Current curricula are largely “process‑based”, requiring a defined amount of exposure, such as a minimum number of lectures or cases [33]. Bedside case teaching provides real clinical scenarios and enhances problem‑solving abilities. Unlike previous approaches, the case teaching method in this study requires students to create cases based on their own clinical assessments, making scenarios more practical and interactive. Furthermore, simulation‑based teaching effectively bridges theory and practice, enhancing nursing students’ clinical competence [34]. These methods should be continued and refined.

Teaching effectiveness evaluation reflects the acceptability of teaching models and identifies students’ learning gaps early for remedial planning [35]. CBE requires a more systematic approach to evaluation [36]. Therefore, combining formative (process) and summative (final) evaluations is essential. Formative assessments—such as attendance tracking and skill operation checks—provide continuous feedback and support performance improvement. In contrast, an outcome‑based curriculum aims to prepare students to achieve predefined, evidence‑based goals. This student‑centered approach allows for locally relevant and innovative teaching methods without compromising patient outcomes [37]. Examples include patient/family service quality feedback and OSCE assessments, which comprehensively evaluate knowledge, skills, and attitudes.Ngozika et al. [38] highlighted the mismatch between classroom-learned theory and clinical practice, attributing it to the poor alignment between course content and clinical departments. This underscores the significance of clinical rotation in assessing new graduate nurses’ readiness for professional practice, especially given the increasing demand for qualified nurses in the healthcare sector. The research department’s rotation and time arrangement was divided into two parts: 38 weeks for CR-related specialties and 18 weeks for other basic-related specialties, totaling 56 weeks. Multidisciplinary collaborative management is crucial for cardiac rehabilitation [39]. To strengthen the integration of theory and clinical practice, it is recommended that graduate students rotate through various departments as much as possible. Rotations in basic specialties like endocrinology, neurology, and respiratory care enable graduate students to assess and handle problems from a holistic health perspective. This allows nursing staff to fully play their leading role in the cardiac rehabilitation team, ensures the continuity of care, provides comprehensive and systematic rehabilitation services for patients.

## Limitations

This study has several limitations. First, the selection of experts from relatively homogeneous backgrounds may have introduced some degree of bias into the research results [40]; therefore, future studies should include more diverse expert panels. Second, the developed teaching framework lacks prospective clinical validation. Although the Delphi method achieved moderate expert consensus, the framework’s effectiveness, feasibility, and impact on student learning outcomes have not yet been tested in real‑world clinical teaching settings. Third, we have not yet applied this teaching system in clinical practice and thus have been unable to evaluate its post‑implementation effectiveness. Future research should focus on the prospective clinical application of this system using rigorous study designs (e.g., pre‑post comparisons or cluster randomized trials) and continue to refine it based on practical feedback.

## Conclusion

Based on an extensive review of literature, the research team utilized the Delphi method to establish a clinical practice teaching system for Nursing Master’s students specializing in cardiac rehabilitation. This system encompasses 5 first-level indicators (teaching objectives, teaching contents, teaching methods, evaluation methods, and department rotation and length), 29 second-level indicators, and 85 third-level indicators. The proper application of this index system is conducive to ensuring the effectiveness of the clinical practice of Nursing Master’s students and enhancing the comprehensive problem-solving abilities of nursing graduate students.

## Supporting information

S1 File The Conducting and Reporting of Delphi Studies checklist guidelines. (DOCX)

S2 File S1. Search strategy. (DOCX)

S3 File S2. Demographic characteristics of participants (N = 22). (DOCX)

S4 File 4 Supplementary materials for clinical practice teaching system. This file contains: S3. Clinical practice training objectives; S4. Clinical practice teaching contents; S5. Evaluation of clinical practice teaching; S6. Clinical practice teaching methods; and S7. Rotation and length of clinical practice departments for MNS graduate students in cardiac rehabilitation.
(DOCX)
