# Supplementary material for: Clinical practice teaching system for MNS cardiac rehabilitation: Delphi consensus
Source: PLoS One. 2026 Jun 25;21(6):e0351886. doi: 10.1371/journal.pone.0351886 (PMC13298731; doi:10.1371/journal.pone.0351886)
Supplement: S2 File — (DOCX) [file pone.0351886.s002.docx]

**Search strategy**

| SEARCH  NO. | SEARCH TERMS AND EXPRESSIONS | RESULTS |
| --- | --- | --- |
| pubmed | | |
| #1 | "cardiac rehabilitation"[MeSH Terms] OR cardiac rehabilitation OR cardiovascular rehabilitation OR heart recovery | 93427 |
| #2 | "course"[MeSH Terms] OR course OR curriculum OR educational program OR training program | 3061404 |
| #3 | "teaching"[MeSH Terms] OR teaching OR instruction OR education OR teaching methods | 2710252 |
| #4 | #1 AND #2 AND #3 | 9001 |
| Web of Science | | |
| #1 | ((TS=("cardiac rehabilitation" OR "cardiovascular rehabilitation" OR "heart rehabilitation" OR "cardiac function recovery")) OR TI=("cardiac rehabilitation" OR "cardiovascular rehabilitation" OR "heart rehabilitation" OR "cardiac function recovery")) OR AB=("cardiac rehabilitation" OR "cardiovascular rehabilitation" OR "heart rehabilitation" OR "cardiac function recovery") and Preprint Citation Index | 15,637 |
| #2 | ((TS=("course" OR "curriculum" OR "educational program" OR "training program" OR "educational course")) OR TI=("course" OR "curriculum" OR "educational program" OR "training program" OR "educational course")) OR AB=("course" OR "curriculum" OR "educational program" OR "training program" OR "educational course") and Preprint Citation Index | 1,734,664 |
| #3 | ((TS=("teaching" OR "instruction" OR "education" OR "teaching strategies" OR "educational methods")) OR TI=("teaching" OR "instruction" OR "education" OR "teaching strategies" OR "educational methods")) OR AB=("teaching" OR "instruction" OR "education" OR "teaching strategies" OR "educational methods") and Preprint Citation Index | 3,648,315 |
| #4 | #1 AND #2 AND #3 | 166 |

| Embase | | |
| --- | --- | --- |
| #1 | 'heart rehabilitation'/exp OR 'cardiac rehabilitation':ti,ab,kw OR 'cardiovascular rehabilitation':ti,ab,kw OR 'heart rehabilitation':ti,ab,kw OR 'cardiac function recovery':ti,ab,kw | 22104 |
| #2 | 'curriculum'/exp OR'course':ti,ab,kw OR 'curriculum':ti,ab,kw OR 'educational program':ti,ab,kw OR 'training program':ti,ab,kw OR 'educational course':ti,ab,kw | 1,141,754 |
| #3 | 'education'/exp OR 'instruction':ti,ab,kw OR 'education':ti,ab,kw OR 'teaching strategies':ti,ab,kw OR 'educational methods':ti,ab,kw | 2,155,331 |
| #4 | #1 AND #2 AND #3 | 278 |
| EBSCO (CINAHL) | | |
| #1 | TI ("cardiac rehabilitation" OR "cardiovascular rehabilitation" OR "heart rehabilitation" OR "cardiac function recovery") OR AB ("cardiac rehabilitation" OR "cardiovascular rehabilitation" OR "heart rehabilitation" OR "cardiac function recovery") OR SU ("cardiac rehabilitation" OR "cardiovascular rehabilitation" OR "heart rehabilitation" OR "cardiac function recovery") | 5051 |
| #2 | TI ("course" OR "curriculum" OR "educational program" OR "training program" OR "nursing curriculum") OR AB ("course" OR "curriculum" OR "educational program" OR "training program" OR "nursing curriculum") OR SU ("course" OR "curriculum" OR "educational program" OR "training program" OR "nursing curriculum") | 195,067 |
| #3 | TI ("teaching" OR "instruction" OR "education" OR "clinical teaching" OR "healthcare instruction") OR AB ("teaching" OR "instruction" OR "education" OR "clinical teaching" OR "healthcare instruction") OR SU ("teaching" OR "instruction" OR "education" OR "clinical teaching" OR "healthcare instruction") | 848,967 |
| #4 | #1 AND #2 AND #3 | 65 |
