# Supplementary material for: Clinical practice teaching system for MNS cardiac rehabilitation: Delphi consensus
Source: PLoS One. 2026 Jun 25;21(6):e0351886. doi: 10.1371/journal.pone.0351886 (PMC13298731; doi:10.1371/journal.pone.0351886)
Supplement: S3 File — (DOCX) [file pone.0351886.s003.docx]

**S2** Demographic characteristics of participants (N = 22)

| **Variables** | **Group** | **Round 1(n=23)** | | **Round 2(n=22)** | |
| --- | --- | --- | --- | --- | --- |
|  |  | **n** | **％** | **n** | **％** |
| Age (years) | 32-42 | 9 | 39.1 | 9 | 40.9 |
|  | 43-53 | 12 | 52.2 | 11 | 50.0 |
|  | ＞53 | 2 | 8.6 | 2 | 9.0 |
| Years of service (years) | 5-15 | 6 | 26.1 | 6 | 27.3 |
|  | 16-26 | 9 | 39.1 | 8 | 36.4 |
|  | ＞26 | 8 | 34.7 | 8 | 36.3 |
| The title of a professional post | Intermediate | 7 | 30.4 | 7 | 31.8 |
|  | Deputy senior | 11 | 47.8 | 10 | 45.5 |
|  | Senior | 5 | 21.7 | 5 | 22.7 |
| Educational background | Undergraduate course | 10 | 43.4 | 10 | 45.5 |
|  | master | 8 | 34.8 | 7 | 31.8 |
|  | Learned scholar | 5 | 21.7 | 5 | 22.7 |
| Professional field | Nursing education | 4 | 17.4 | 4 | 18.2 |
|  | Nursing management | 3 | 13.0 | 3 | 13.6 |
|  | Clinical nursing of cardiac rehabilitation | 15 | 65.2 | 14 | 63.6 |
|  | Clinical care of cardiac rehabilitation | 1 | 4.3 | 1 | 4.5 |
