# Supplementary material for: Clinical practice teaching system for MNS cardiac rehabilitation: Delphi consensus
Source: PLoS One. 2026 Jun 25;21(6):e0351886. doi: 10.1371/journal.pone.0351886 (PMC13298731; doi:10.1371/journal.pone.0351886)
Supplement: S4 File — This file contains: S3. Clinical practice training objectives; S4. Clinical practice teaching contents; S5. Evaluation of clinical practice teaching; S6. Clinical practice teaching methods; and S7. Rotation and length of clinical practice departments for MNS graduate students in cardiac rehabilitation. (DOCX) [file pone.0351886.s004.docx]

**Supplementary Table S3.** Clinical practice training objectives of MNS graduate students in cardiac rehabilitation

| **Indicators** | **Rating of importance** | **Coefficient of variation** | **Weighted value** |
| --- | --- | --- | --- |
| **Teaching objective** | **4.95±0.21** | **0.04** | **0.016** |
| **Knowledge objective** | **5.00±0.00** | **0.00** | **0.016** |
| State the basic principles for formulating nutritional prescriptions for cardiovascular diseases | 4.73±0.46 | 0.10 | 0.005 |
| List dietary and nutritional guidance methods for common cardiovascular diseases and their risk factors | 4.77±0.43 | 0.09 | 0.006 |
| Apply common nursing interventions and cognitive-behavioral techniques to implement basic psychosocial interventions | 4.55±0.60 | 0.13 | 0.002 |
| Identify and differentiate the severity of common mental health issues in cardiovascular disease patients and determine referral criteria | 4.73±0.46 | 0.10 | 0.003 |
| List the content, procedures, key points, and significance of cardiopulmonary rehabilitation assessments (e.g., biological/medical history evaluation, metabolic abnormality assessment, physical fitness assessment, activities of daily living evaluation, cardiopulmonary exercise testing risk assessment, and mental/psychological evaluation) | 4.95±0.21 | 0.04 | 0.016 |
| Interpret cardiopulmonary rehabilitation risk assessment results to guide the development of optimal cardiac rehabilitation care plans | 5.00±0.00 | 0.00 | 0.016 |
| Describe the types, efficacy, principles, and precautions of exercise therapy for cardiovascular disease patients | 4.82±0.40 | 0.08 | 0.013 |
| Outline key indicators for exercise training prescriptions (e.g., aerobic, resistance, flexibility, balance, and coordination exercises) in cardiovascular disease patients | 4.91±0.29 | 0.06 | 0.016 |
| Outline key indicators for exercise training prescriptions (e.g., aerobic, resistance, flexibility, balance, and coordination exercises) in cardiovascular disease patients | 4.59±0.59 | 0.13 | 0.005 |
| Summarize the nursing and rehabilitation essentials for patients with common cardiovascular diseases | 4.82±0.40 | 0.08 | 0.013 |
| **Indicators** | **Rating of importance** | **Coefficient of variation** | **Weighted value** |
| **1.2 Competency/Skill Objectives** | **5.00 ±0.00** | **0.00** | **0.016** |
| Accurately distinguish between normal and abnormal electrocardiograms (ECGs) and identify ECG waveforms associated with common cardiovascular diseases |  |  |  |
| Guide cardiovascular disease patients on dietary content, portion sizes, food types, and preparation methods using nutritional management knowledge |  |  |  |
| Implement basic psychosocial interventions by applying common nursing practices and cognitive-behavioral techniques | 4.55±0.60 | 0.13 | 0.004 |
| Recognize and triage the severity of mental health issues in cardiovascular disease patients and determine referral criteria | 4.68±0.57 | 0.12 | 0.003 |
| Interpret cardiopulmonary rehabilitation assessment results to guide cardiac rehabilitation | 4.86±0.35 | 0.07 | 0.009 |
| Design and apply exercise prescriptions tailored to patients with different risk levels | 4.86±0.35 | 0.07 | 0.013 |
| Instruct cardiovascular disease patients on home-based self-care using family-centered cardiac rehabilitation strategies | 4.86±0.35 | 0.07 | 0.009 |
| Identify, assess, and address common nursing problems in cardiovascular disease patients | 4.91±0.29 | 0.06 | 0.013 |
| Proficiently apply cardiac-specific rehabilitation protocols to guide patient training | 4.91±0.29 | 0.06 | 0.016 |
| Operate cardiopulmonary assessment, monitoring, and rehabilitation equipment with proficiency and interpret critical parameter indicators | 4.82±0.40 | 0.08 | 0.013 |
| Demonstrate foundational competency in addressing and resolving complex clinical cardiac-related nursing challenges | 4.82±0.40 | 0.08 | 0.009 |
| **1.3 Professionalism Objectives** | **4.86 ±0.35** | **0.07** | **0.009** |
| Cultivate proper professional values and a sense of identity in the nursing field | 4.73±0.46 | 0.10 | 0.005 |
| Embrace patient-centered care and humanitarian principles | 4.73±0.46 | 0.10 | 0.005 |
| Uphold professional ethics and adhere to legal nursing practices | 4.91±0.29 | 0.06 | 0.013 |
|  |  |  |  |
| **Indicators** | **Rating of importance** | **Coefficient of variation** | **Weighted value** |
| Foster interdisciplinary collaboration and teamwork awareness | 4.86±0.35 | 0.07 | 0.013 |
| Develop risk assessment awareness and multidimensional evaluation competencies | 4.82±0.40 | 0.08 | 0.013 |

**Supplementary Table S4.Clinical practice teaching contents of MNS graduate students in cardiac rehabilitation**

| **Indicators** | **Rating of importance** | **Coefficient of variation** | **Weighted**  **value** |
| --- | --- | --- | --- |
| **Teaching content** | **4.91±0.29** | **0.06** | **0.009** |
| **Specialized Nursing Care for Cardiovascular Disease Patients** | **4.91±0.29** | **0.06** | **0.009** |
| Postoperative Care for Percutaneous Coronary Stent Implantation | 4.95±0.21 | 0.04 | 0.013 |
| Nursing Care for Chronic Heart Failure | 4.95±0.21 | 0.04 | 0.013 |
| Nursing Care for Stable Angina Pectoris | 4.86±0.35 | 0.07 | 0.009 |
| Nursing Care for Arrhythmia | 4.95±0.21 | 0.04 | 0.013 |
| Nursing Care for Myocardial Infarction | 4.95±0.22 | 0.04 | 0.013 |
| Postoperative Care for Pacemaker Implantation | 4.95±0.22 | 0.04 | 0.013 |
| Postoperative Care for Coronary Artery Bypass Grafting | 4.95±0.21 | 0.04 | 0.013 |
| Postoperative Care for Patients Following Heart Valve Replacement Surgery | 4.95±0.21 | 0.04 | 0.013 |
| Postoperative Care for Heart Transplantation | 4.91±0.29 | 0.06 | 0.009 |
| Nursing Care for Aortic Dissection | 4.95±0.21 | 0.04 | 0.013 |
| Nursing Care for Diabetic Patients | 4.64±0.49 | 0.11 | 0.003 |
| Nursing Care for Stroke Patients | 4.64±0.49 | 0.11 | 0.002 |
| Nursing Care for Pneumonia Patients | 4.41±0.67 | 0.15 | ＜0.001 |
| Nursing Care for Chronic Obstructive Pulmonary Emphysema | 4.50±0.67 | 0.14 | 0.001 |
| **Cardiac Emergency Techniques** | **4.95±0.21** | **0.04** | **0.013** |
| Emergency Airway Management Techniques | 4.36±0.85 | 0.19 | 0.001 |
| Electrocardiographic (ECG) Monitoring Techniques | 4.86±0.35 | 0.07 | 0.006 |
| Electrical Defibrillation Techniques | 4.82±0.40 | 0.08 | 0.005 |
| Mechanical Ventilator Application Techniques | 4.59±0.50 | 0.11 | 0.003 |
| Circulatory System Hemodynamic Monitoring Techniques | 4.50±0.67 | 0.14 | 0.002 |
| Cardiopulmonary Resuscitation (CPR) | 4.77±0.43 | 0.09 | 0.005 |
| Airway Management Techniques | 4.59±0.67 | 0.15 | 0.002 |
| **Cardiac rehabilitation assessment and training** | **5.00±0.00** | **0.00** | **0.016** |
| Exercise Electrocardiogram Testing (treadmill exercise or treadmill exercise) | 4.82±0.40 | 0.08 | 0.009 |
| Cardiopulmonary Exercise Testing (CPET) | 4.91±0.29 | 0.06 | 0.016 |
| 6-Minute Walk Test | 4.95±0.21 | 0.04 | 0.016 |
| **Indicators** | **Rating of importance** | **Coefficient of variation** | **Weighted**  **value** |
| Enhanced External Counterpulsation Therapy | 4.77±0.43 | 0.09 | 0.005 |
| Cardiac Function Grading and Assessment | 4.91±0.29 | 0.06 | 0.013 |
| Subjective Symptom Assessment for Daily Activities | 4.86±0.35 | 0.07 | 0.019 |
| Biological/Medical History Evaluation | 4.77±0.43 | 0.09 | 0.013 |
| Quality of Life Assessment | 4.86±0.35 | 0.07 | 0.009 |
| Body Positioning Techniques for Cardiopulmonary Rehabilitation | 4.91±0.29 | 0.06 | 0.013 |
| Bedside Training Methods (Sitting, Standing, Stepping, Walking) | 4.95±0.21 | 0.04 | 0.013 |
| Traditional Chinese Medicine (TCM) Techniques for Cardiopulmonary Rehabilitation Nursing | 4.18±0.66 | 0.16 | ＜0.001 |
| **Exercise Endurance, Risk Assessment, and Rehabilitation Techniques** | **5.00±0.00** | **0.00** | **0.016** |
| Maximal Strength Assessment (Resistance) | 4.68±0.57 | 0.12 | 0.009 |
| Chair Sit-and-Reach Test (Flexibility) | 4.68±0.57 | 0.12 | 0.009 |
| Back Scratch Test (Flexibility) | 4.71±0.56 | 0.12 | 0.009 |
| Single-Leg Stance Balance Test (Balance) | 4.71±0.56 | 0.12 | 0.009 |
| 2.4-Meter Up-and-Go Test (Balance) | 4.77±0.53 | 0.11 | 0009 |
| Warm-Up and Cool-Down Techniques | 4.77±0.53 | 0.11 | 0.007 |
| Upper and Lower Limb Active Movement Training | 4.73±0.55 | 0.12 | 0.006 |
| Dumbbell/Resistance Band Rehabilitation Training | 4.77±0.53 | 0.11 | 0.006 |
| Flexibility Training Techniques | 4.73±0.55 | 0.12 | 0.006 |
| Muscle Relaxation Training Techniques | 4.73±0.55 | 0.11 | 0.006 |
| Breathing Training Techniques | 4.73±0.55 | 0.11 | 0.016 |
| Pursed-Lip Breathing Training | 4.91±0.43 | 0.09 | 0.013 |
| **Psychological Assessment and Guidance** | **4.86±0.47** | **0.10** | **0.003** |
| Anxiety and Depression Screening and Scoring | 4.64±0.49 | 0.11 | 0.006 |
| Intervention and Guidance for Patients with Anxiety/Depression | 4.77±0.53 | 0.11 | 0.003 |
| **Nutritional Assessment and Dietary Guidance** | **4.68±0.57** | **0.12** | **0.013** |
| Metabolic Abnormality Evaluation | 4.91±0.29 | 0.06 | 0.001 |
| Nutritional Prescription Development for Cardiovascular Disease Patients | 4.55±0.67 | 0.15 | 0.003 |
| Dietary Guidance for Cardiovascular Disease Patients | 4.68±0.57 | 0.12 | 0.004 |

**Supplementary Table S5. Evaluation of clinical practice teaching of MNS graduate students in cardiac rehabilitation**

| **Indicators** | **Rating of importance** | **Coefficient of variation** | **Weighted value** |
| --- | --- | --- | --- |
| **Teaching evaluation** | **4.68±0.48** | **0.10** | **0.007** |
| **Formative Evaluation** | **4.68±0.48** | **0.10** | **0.007** |
| Department Attendance Tracking | 4.73±0.55 | 0.12 | 0.006 |
| End-of-Rotation Assessment (Theory + Practical Skills) | 4.82±0.40 | 0.08 | 0.009 |
| Evaluation of Skill Procedure Completion Volume | 4.73±0.46 | 0.10 | 0.006 |
| Evaluation of Case Nursing Completion Volume | 4.73±0.46 | 0.10 | 0.009 |
| Rehabilitation Nursing Case Report | 4.91±0.29 | 0.06 | 0.016 |
| Participation Engagement Assessment (e.g., lectures, ward rounds, case discussions) | 4.82±0.40 | 0.08 | 0.013 |
| Nursing Documentation Quality Evaluation | 4.82±0.50 | 0.10 | 0.009 |
| **Summative Evaluation** | **4.68±0.48** | **0.10** | **0.007** |
| Patient/Family Service Quality Feedback | 4.73±0.63 | 0.13 | 0.013 |
| Departmental Reflection Report (Self-Evaluation) | 4.68±0.65 | 0.14 | 0.009 |
| OSCE Assessment (Rehabilitation Evaluation, Case Defense, Common Cardiac Rehabilitation Techniques, and Nursing Skill Proficiency) | 4.82±0.40 | 0.08 | 0.013 |

**Supplementary Table S6. Clinical practice teaching methods of MNS graduate students in cardiac rehabilitation**

| **Indicators** | **Rating of importance** | **Coefficient of variation** | **Weighted value** |
| --- | --- | --- | --- |
| **Teaching method** | **4.77±0.53** | **0.11** | **0.006** |
| Bedside Case-Based Teaching | 4.77±0.43 | 0.11 | 0.013 |
| Departmental Mini-Lectures | 4.91±0.29 | 0.06 | 0.009 |
| Nursing Case Analysis and Discussion | 4.73±0.46 | 0.10 | 0.013 |
| Nursing Rounds and Case Presentations | 4.86±0.35 | 0.07 | 0.009 |
| Thematic Seminars and Literature Reports | 4.64±0.58 | 0.13 | 0.005 |
| Academic Conferences and Special Lectures | 4.73±0.46 | 0.10 | 0.009 |
| Demonstration and Simulation Techniques | 4.86±0.35 | 0.07 | 0.009 |

**Supplementary Table S7. Rotation and length of clinical practice departments of MNS graduate students in cardiac rehabilitation direction**

| **Indicators** |  | **Rating of importance** | **Coefficient of variation** | **Weighted value** |
| --- | --- | --- | --- | --- |
| **Rotation of departments in time** |  | **4.91±0.29** | **0.06** | **0.009** |
| Cardiology Department | 8 weeks | 4.77±0.53 | 0.11 | 0.003 |
| Cardiothoracic Surgery Department | 8 weeks | 4.68±0.57 | 0.12 | 0.003 |
| Cardiac Intensive Care Unit | 2 weeks | 4.55±0.67 | 0.15 | 0.004 |
| Cardiothoracic Surgery Intensive Care Unit (CSICU) | 2 weeks | 4.68±0.57 | 0.12 | 0.004 |
| Cardiac Rehabilitation Center | 10 weeks | 4.77±0.53 | 0.11 | ＜0.001 |
| Rehabilitation Medicine Department | 8 weeks | 4.36±0.73 | 0.17 | 0.001 |
| Psychiatry/Psychology Department | 4weeks | 4.36±0.79 | 0.18 | 0.001 |
| Nutrition department | 4 weeks | 4.41±0.80 | 0.18 | 0.001 |
| Other Relevant Departments (Endocrinology) | 4 weeks | 4.32±0.84 | 0.19 | 0.002 |
| Traditional Chinese Medicine (TCM) Department | 2 weeks | 4.23±0.69 | 0.16 | 0.001 |
