# Supplementary material for: Clinical practice teaching system for MNS cardiac rehabilitation: Delphi consensus
Source: PLoS One. 2026 Jun 25;21(6):e0351886. doi: 10.1371/journal.pone.0351886 (PMC13298731; doi:10.1371/journal.pone.0351886)
Supplement: Attachment — Submitted filename: Response to Reviewers.docx [file pone.0351886.s006.docx]

Response to Reviewers

Manuscript ID: PONE-D-25-36678

Title: Clinical Practice Teaching System for MNS Cardiac Rehabilitation: Delphi Consensus

Journal: PLOS ONE

To the Academic Editor, Dr. Yagnik Dave, and the Reviewer,

We sincerely thank you for the constructive feedback and the opportunity to revise our manuscript. We have carefully considered all comments from the editor and the reviewer and have made comprehensive revisions to address each point. All changes have been incorporated into the revised manuscript. Below, we provide a point-by-point response to the comments.

Journal Requirements (Editor’s Comments):

1. Style Requirements:

Please ensure that your manuscript meets PLOS ONE's style requirements...

**Author Response**: We have thoroughly reformatted the entire manuscript using the official PLOS ONE style templates provided (for the main body and title/authors/affiliations). We confirm that the manuscript now complies with all journal formatting guidelines, including file naming conventions.

**The key formatting updates include:**

Title & Authorship Block:

The manuscript title has been formatted as per journal style.

All author academic titles and honorifics have been removed from the author list, presenting only names and affiliations.

The corresponding author has been clearly designated using the journal’s specified symbol (*), and the email address has been formatted according to the required standard.

**Figures, Tables, and Supporting Information:**

All tables and figures have been reviewed. Their placement and citations within the text have been ensured to follow journal guidelines (e.g., each is now cited in sequential order in the main text before it is presented).

These comprehensive formatting changes have been applied throughout the manuscript to align with the provided templates and submission guidelines.

**2. Data Availability Statement:**

In the online submission form you indicate that your data is not available for proprietary reasons... Please revise your data statement to a non-author institutional point of contact...

**Author Response:** We sincerely apologize for the initial non-compliant data statement. Following the journal’s policy and the reviewer’s recommendation, we have taken the following actions:

We have deposited all underlying data supporting the findings of this Delphi study (including anonymized survey response datasets, consensus tables, and statistical analysis files) in the public repository Figshare.

The dataset has been assigned a Digital Object Identifier (DOI), ensuring permanent and unrestricted access.

We have completely rewritten the Data Availability Statement in the manuscript to provide the direct repository link, as required.

The updated statement no longer lists an author as the contact point. The data is now publicly accessible to anyone via the DOI.

**The updated Data Availability Statement in the manuscript reads:**

“All relevant data underlying the findings of this Delphi consensus study, including anonymized participant responses, item-level statistics, and final consensus tables, are fully available without restriction from the Figshare repository: **[https://doi.org/10.6084/m9.figshare.31310665]**

We have also sent the full text of this updated statement to the editorial office via separate email, as requested.

**3. Supporting Information Captions:**

Please include captions for your Supporting Information files...

**Author Response**: We have added a dedicated “Supporting Information” section at the end of the manuscript, immediately following the References. This section lists the full captions for all supporting files (e.g.,Table 1. Search strategy; Table 2 Demographic characteristics of participants). All in-text citations to these files have been verified and updated to match.

**4. & 5. References:**

If the reviewer comments include a recommendation to cite specific works... Please review your reference list...

**Author Response**: We have carefully reviewed the reference list for completeness, accuracy, and to ensure no retracted papers are cited. While the reviewer did not suggest specific citations, we have verified the currency and relevance of all references. Any minor formatting adjustments needed to align with PLOS ONE style have been made. These updates are reflected in the revised manuscript.

**Reviewer #1’s Comments:**

We thank Reviewer #1 (Dr. Hemangkumar Sudhakarbhai Jani) for the positive assessment of our work and the crucial, actionable feedback regarding data availability.

**General Comment:**

“The study addresses an important educational gap... The methodology and interpretation are appropriate, and the manuscript is generally clear and coherent.”

**Author Response**: We are grateful for the reviewer’s positive and encouraging assessment of our work’s significance and execution.

**Specific Major Comment:**

“Data Availability Concern: The statement ‘data available from the corresponding author upon request’ does not comply with the PLOS ONE Data Policy... Authors should deposit all underlying data... in a public repository (e.g., Figshare, Dryad) and update the Data Availability Statement...”

**Author Response**: We fully agree with the reviewer on the importance of data transparency and reproducibility. As detailed in our response to the editor’s point #2 above, we have acted upon this essential recommendation. We have deposited the complete dataset in Figshare, a recommended public repository, and have updated the Data Availability Statement in the manuscript to provide the direct, permanent DOI link. This ensures full, unrestricted access to the data, significantly enhancing the study’s transparency and compliance with journal standards.

**Additional Checks:**

“Is the manuscript presented in an intelligible fashion and written in standard English?”

**Author Response**: Following the reviewer’s confirmation that the manuscript is clear, we have nonetheless conducted a thorough proofreading of the revised text to correct any minor typographical or grammatical errors, further improving the clarity of the presentation.

We believe that our revisions have comprehensively addressed all concerns raised. We are grateful for the time and expertise invested in evaluating our manuscript and hope that the revised version now meets the high standards of PLOS ONE for publication.

Sincerely,

**Yanyan Fan*

Binzhou Medical University

Addr: Binzhou Medical University, No. 346 Guanhai Road, Binhai Road Street, Yantai City, Shandong Province, China

E-mail: fanyanyan@bzmc.edu.cn

**Guiyun Wang*

Shandong Xiehe University

Addr: Shandong Xiehe University, No. 6277, Jiqing Road, Licheng District, Jinan City, Shandong Province, China

E-mail: [wangguiyun@sdxiehe.edu.cn](mailto:wangguiyun@sdxiehe.edu.cn)
