# Supplementary material for: Clinical practice teaching system for MNS cardiac rehabilitation: Delphi consensus
Source: PLoS One. 2026 Jun 25;21(6):e0351886. doi: 10.1371/journal.pone.0351886 (PMC13298731; doi:10.1371/journal.pone.0351886)
Supplement: Attachment — Submitted filename: Response_to_Reviewers_auresp_2.docx [file pone.0351886.s007.docx]

Response to Reviewers

Manuscript ID: PONE-D-25-36678

Title: Clinical Practice Teaching System for MNS Cardiac Rehabilitation: Delphi Consensus

Journal: PLOS ONE

To the Academic Editor, Dr. Yagnik Dave, and the Reviewer,

We thank you for the thoughtful and constructive comments on our manuscript. We have carefully addressed all the points raised by the academic editor and reviewers. Below we provide a point‑by‑point response. All changes in the manuscript have been highlighted in the “Revised Manuscript with Track Changes” file.

Journal Requirements (Editor’s Comments):

**Comment 1**

“Tone down claims of ‘strong consensus’ and revise to reflect moderate agreement (as per Kendall’s W values).”

**Response**: We agree with the reviewer. The Kendall’s W values ranged from 0.13 to 0.21, which indicates moderate, not strong, agreement among experts. Accordingly, we have revised the manuscript as follows:

Abstract, Results section:

Original: “The Delphi study demonstrated high engagement … with robust validity (Cr=0.83-0.84; Kendall's W=0.13-0.21, p<0.05). Consensus yielded a structured framework…”

Revised to: “The Delphi study demonstrated high engagement … and acceptable expert authority (Cr = 0.83‑0.84). Kendall’s W values ranged from 0.13 to 0.21 (p < 0.05), indicating moderate agreement among experts. The consensus process yielded a structured framework…”

Results, “Results of the First Delphi Survey”:

Original: “The second Delphi round confirmed unanimous expert consensus on all items…”

Revised to: “The second Delphi round confirmed consistent expert agreement on all items…”

No other statements implying “strong consensus” remain in the manuscript.

**Comment 2**

“Improve English language quality (grammar, clarity, and reduce repetition in Discussion).”

**Response**: We have thoroughly revised the Discussion section to improve grammar, clarity, and conciseness. Major changes include:

Corrected grammatical errors (e.g., “This study is kind to pinpoint” → “This study identified”).

Removed redundant content (e.g., duplicate lists of interventions such as nutrition counseling, weight management, etc.).

Simplified overly complex sentences and improved logical flow.

Clarified ambiguous pronoun references (e.g., “which encourages medical schools” → “Medical schools should integrate…”).

Reorganized the Discussion into clearer thematic paragraphs.

The revised Discussion is now more readable and free of repetition. All changes are visible in the “Revised Manuscript with Track Changes” file.

**Comment 3**

“Clarify ethical statement—Delphi studies involve human participants; include IRB approval or exemption with justification and reference number.”

**Response**: We thank the reviewer for this important correction. The original statement (“does not involve human experiments”) was inaccurate. We have revised the Ethical Considerations section as follows:

“This study was conducted in accordance with the Declaration of Helsinki (as revised in 2013). The research involved human participants (expert panel members) but did not include patients, clinical interventions, or any identifiable sensitive data. According to the policy of the Institutional Review Board of Binzhou Medical University, formal ethics approval is not required for studies that solely collect anonymous expert opinions through surveys without intervention or patient contact. Therefore, this study was exempt from full ethical review. Nevertheless, all participating experts provided electronic informed consent prior to completing the questionnaire. They were fully informed of the study purpose, the voluntary nature of participation, and their right to withdraw at any time without negative consequences. Anonymity and confidentiality were strictly maintained throughout the study. (No reference number was issued under this exemption; written confirmation from the IRB can be provided upon request.)”

Thus, we now explicitly acknowledge human participation, provide the exemption justification based on institutional policy, and note the absence of a reference number (while offering to provide IRB confirmation if needed).

**Comment 4**

“Add a clear limitation stating that the framework lacks prospective clinical validation.”

**Response**: We agree and have added this limitation. In the Limitations section, we now state:

“Second, the developed teaching framework lacks prospective clinical validation. Although the Delphi method achieved moderate expert consensus, the framework’s effectiveness, feasibility, and impact on student learning outcomes have not yet been tested in real‑world clinical teaching settings.”

We also revised the future research direction accordingly: “Future research should focus on the prospective clinical application of this system using rigorous study designs (e.g., pre‑post comparisons or cluster randomized trials).”

The full revised Limitations paragraph is included in the track‑changed manuscript.

**Comment 5**

“Consider moving lengthy Tables (3–7) to Supplementary Material to improve readability.”

**Response**: We agree with the reviewer. To improve the readability of the main manuscript, we have:

Removed the full Tables 3, 4, 5, 6, and 7 from the Results section.

Added a sentence in the “Results of the First Delphi Survey” paragraph: “The detailed indicator lists with importance ratings, coefficients of variation, and weights are provided in Supplementary Tables S1–S5 (see Supplementary File 1).”

Created a separate Supplementary Material file containing Supplementary Tables S1–S5 (which correspond to original Tables 3–7).

Retained Tables 1 and 2 (search strategy and participant demographics) in the main text, as they are short and essential for understanding.

This change significantly improves the flow of the manuscript while preserving all detailed data for interested readers.

We believe the revised manuscript is substantially improved and hope it now meets the journal’s standards for publication. We appreciate your time and expertise in reviewing this work.

Sincerely,

**Yanyan Fan*

Binzhou Medical University

Addr: Binzhou Medical University, No. 346 Guanhai Road, Binhai Road Street, Yantai City, Shandong Province, China

E-mail: fanyanyan@bzmc.edu.cn

**Guiyun Wang*

Shandong Xiehe University

Addr: Shandong Xiehe University, No. 6277, Jiqing Road, Licheng District, Jinan City, Shandong Province, China

E-mail: [wangguiyun@sdxiehe.edu.cn](mailto:wangguiyun@sdxiehe.edu.cn)
