# Supplementary material for: Clinical practice teaching system for MNS cardiac rehabilitation: Delphi consensus
Source: PLoS One. 2026 Jun 25;21(6):e0351886. doi: 10.1371/journal.pone.0351886 (PMC13298731; doi:10.1371/journal.pone.0351886)
Supplement: Peer review history

# Peer review: Decision Letter 0

**DOI:** 10.1371/journal.pone.0351886.r001

**Authors:** Yagnik Dave

7 Jan 2026

PONE-D-25-36678

Clinical Practice Teaching System for MNS Cardiac Rehabilitation: Delphi Consensus

PLOS One

Dear Dr. wang,

Thank you for submitting your manuscript to PLOS ONE. After careful consideration, we feel that it has merit but does not fully meet PLOS ONE’s publication criteria as it currently stands. Therefore, we invite you to submit a revised version of the manuscript that addresses the points raised during the review process.

Please submit your revised manuscript by Feb 21 2026 11:59PM. If you will need more time than this to complete your revisions, please reply to this message or contact the journal office at plosone@plos.org. When you're ready to submit your revision, log on to https://www.editorialmanager.com/pone/ and select the 'Submissions Needing Revision' folder to locate your manuscript file.

Please include the following items when submitting your revised manuscript:

- A letter that responds to each point raised by the academic editor and reviewer(s). You should upload this letter as a separate file labeled 'Response to Reviewers'.
- A marked-up copy of your manuscript that highlights changes made to the original version. You should upload this as a separate file labeled 'Revised Manuscript with Track Changes'.
- An unmarked version of your revised paper without tracked changes. You should upload this as a separate file labeled 'Manuscript'.

-->If you would like to make changes to your financial disclosure, please include your updated statement in your cover letter. Guidelines for resubmitting your figure files are available below the reviewer comments at the end of this letter.

If applicable, we recommend that you deposit your laboratory protocols in protocols.io to enhance the reproducibility of your results. Protocols.io assigns your protocol its own identifier (DOI) so that it can be cited independently in the future. For instructions see: https://journals.plos.org/plosone/s/submission-guidelines#loc-laboratory-protocols. Additionally, PLOS ONE offers an option for publishing peer-reviewed Lab Protocol articles, which describe protocols hosted on protocols.io. Read more information on sharing protocols at https://plos.org/protocols?utm_medium=editorial-email&utm_source=authorletters&utm_campaign=protocols.

We look forward to receiving your revised manuscript.

Kind regards,

Yagnik Dave

Academic Editor

PLOS One

**Journal Requirements:**

When submitting your revision, we need you to address these additional requirements.

1. Please ensure that your manuscript meets PLOS ONE's style requirements, including those for file naming. The PLOS ONE style templates can be found at

https://journals.plos.org/plosone/s/file?id=wjVg/PLOSOne_formatting_sample_main_body.pdf and

https://journals.plos.org/plosone/s/file?id=ba62/PLOSOne_formatting_sample_title_authors_affiliations.pdf

2. In the online submission form you indicate that your data is not available for proprietary reasons and have provided a contact point for accessing this data. Please note that your current contact point is a co-author on this manuscript. According to our Data Policy, the contact point must not be an author on the manuscript and must be an institutional contact, ideally not an individual. Please revise your data statement to a non-author institutional point of contact, such as a data access or ethics committee, and send this to us via return email. Please also include contact information for the third party organization, and please include the full citation of where the data can be found.

3. Please include captions for your Supporting Information files at the end of your manuscript, and update any in-text citations to match accordingly. Please see our Supporting Information guidelines for more information: http://journals.plos.org/plosone/s/supporting-information.

4. If the reviewer comments include a recommendation to cite specific previously published works, please review and evaluate these publications to determine whether they are relevant and should be cited. There is no requirement to cite these works unless the editor has indicated otherwise.

5. Please review your reference list to ensure that it is complete and correct. If you have cited papers that have been retracted, please include the rationale for doing so in the manuscript text, or remove these references and replace them with relevant current references. Any changes to the reference list should be mentioned in the rebuttal letter that accompanies your revised manuscript. If you need to cite a retracted article, indicate the article’s retracted status in the References list and also include a citation and full reference for the retraction notice.

Additional Editor Comments:

Minor revision is required to fulfill the journal guideline.

[Note: HTML markup is below. Please do not edit.]

Reviewers' comments:

Reviewer's Responses to Questions

**Comments to the Author**

1. Is the manuscript technically sound, and do the data support the conclusions?

The manuscript must describe a technically sound piece of scientific research with data that supports the conclusions. Experiments must have been conducted rigorously, with appropriate controls, replication, and sample sizes. The conclusions must be drawn appropriately based on the data presented.

Reviewer #1: Yes

**********

2. Has the statistical analysis been performed appropriately and rigorously?

Reviewer #1: Yes

**********

3. Have the authors made all data underlying the findings in their manuscript fully available?

The PLOS Data policy requires authors to make all data underlying the findings described in their manuscript fully available without restriction, with rare exception (please refer to the Data Availability Statement in the manuscript PDF file). The data should be provided as part of the manuscript or its supporting information, or deposited to a public repository. For example, in addition to summary statistics, the data points behind means, medians and variance measures should be available. If there are restrictions on publicly sharing data—e.g. participant privacy or use of data from a third party—those must be specified.

Reviewer #1: No

**********

4. Is the manuscript presented in an intelligible fashion and written in standard English?

PLOS ONE does not copyedit accepted manuscripts, so the language in submitted articles must be clear, correct, and unambiguous. Any typographical or grammatical errors should be corrected at revision, so please note any specific errors here.

Reviewer #1: Yes

**********

5. Review Comments to the Author

Please use the space provided to explain your answers to the questions above. You may also include additional comments for the author, including concerns about dual publication, research ethics, or publication ethics. (Please upload your review as an attachment if it exceeds 20,000 characters.)

Reviewer #1: The study addresses an important educational gap in cardiac rehabilitation nursing and presents a well-structured Delphi-based framework.

The methodology and interpretation are appropriate, and the manuscript is generally clear and coherent.

Data Availability Concern: The statement “data available from the corresponding author upon request” does not comply with the PLOS ONE Data Policy, which requires unrestricted access.

Authors should deposit all underlying data (e.g., Delphi survey results, consensus tables, statistical analyses) in a public repository (e.g., Figshare, Dryad) and update the Data Availability Statement with a repository link or DOI.

Ensuring open access to data will enhance the study’s transparency, reproducibility, and compliance with journal standards.

**********

6. PLOS authors have the option to publish the peer review history of their article (what does this mean?). If published, this will include your full peer review and any attached files.

If you choose “no”, your identity will remain anonymous but your review may still be made public.

**Do you want your identity to be public for this peer review?**   For information about this choice, including consent withdrawal, please see our Privacy Policy.

Reviewer #1: **Yes:**  Dr Hemangkumar Sudhakarbhai Jani

**********

[NOTE: If reviewer comments were submitted as an attachment file, they will be attached to this email and accessible via the submission site. Please log into your account, locate the manuscript record, and check for the action link "View Attachments." If this link does not appear, there are no attachment files.]

To ensure your figures meet our technical requirements, please review our figure guidelines: https://journals.plos.org/plosone/s/figures

You may also use PLOS’s free figure tool, NAAS, to help you prepare publication quality figures: https://journals.plos.org/plosone/s/figures#loc-tools-for-figure-preparation.

NAAS will assess whether your figures meet our technical requirements by comparing each figure against our figure specifications.

# Author comment: Author response to Decision Letter 1

**DOI:** 10.1371/journal.pone.0351886.r002

20 Apr 2026

We sincerely thank you for the constructive feedback and the opportunity to revise our manuscript. We have carefully considered all comments from the editor and the reviewer and have made comprehensive revisions to address each point. All changes have been incorporated into the revised manuscript. Below, we provide a point-by-point response to the comments.

Journal Requirements (Editor’s Comments):

1. Style Requirements:

Please ensure that your manuscript meets PLOS ONE's style requirements...

Author Response: We have thoroughly reformatted the entire manuscript using the official PLOS ONE style templates provided (for the main body and title/authors/affiliations). We confirm that the manuscript now complies with all journal formatting guidelines, including file naming conventions.

The key formatting updates include:

Title & Authorship Block:

The manuscript title has been formatted as per journal style.

All author academic titles and honorifics have been removed from the author list, presenting only names and affiliations.

The corresponding author has been clearly designated using the journal’s specified symbol (*), and the email address has been formatted according to the required standard.

Figures, Tables, and Supporting Information:

All tables and figures have been reviewed. Their placement and citations within the text have been ensured to follow journal guidelines (e.g., each is now cited in sequential order in the main text before it is presented).

These comprehensive formatting changes have been applied throughout the manuscript to align with the provided templates and submission guidelines.

2. Data Availability Statement:

In the online submission form you indicate that your data is not available for proprietary reasons... Please revise your data statement to a non-author institutional point of contact...

Author Response: We sincerely apologize for the initial non-compliant data statement. Following the journal’s policy and the reviewer’s recommendation, we have taken the following actions:

We have deposited all underlying data supporting the findings of this Delphi study (including anonymized survey response datasets, consensus tables, and statistical analysis files) in the public repository Figshare.

The dataset has been assigned a Digital Object Identifier (DOI), ensuring permanent and unrestricted access.

We have completely rewritten the Data Availability Statement in the manuscript to provide the direct repository link, as required.

The updated statement no longer lists an author as the contact point. The data is now publicly accessible to anyone via the DOI.

The updated Data Availability Statement in the manuscript reads:

“All relevant data underlying the findings of this Delphi consensus study, including anonymized participant responses, item-level statistics, and final consensus tables, are fully available without restriction from the Figshare repository: [https://doi.org/10.6084/m9.figshare.31310665]

We have also sent the full text of this updated statement to the editorial office via separate email, as requested.

3. Supporting Information Captions:

Please include captions for your Supporting Information files...

Author Response: We have added a dedicated “Supporting Information” section at the end of the manuscript, immediately following the References. This section lists the full captions for all supporting files (e.g.,Table 1. Search strategy; Table 2 Demographic characteristics of participants). All in-text citations to these files have been verified and updated to match.

4. & 5. References:

If the reviewer comments include a recommendation to cite specific works... Please review your reference list...

Author Response: We have carefully reviewed the reference list for completeness, accuracy, and to ensure no retracted papers are cited. While the reviewer did not suggest specific citations, we have verified the currency and relevance of all references. Any minor formatting adjustments needed to align with PLOS ONE style have been made. These updates are reflected in the revised manuscript.

Reviewer #1’s Comments:

We thank Reviewer #1 (Dr. Hemangkumar Sudhakarbhai Jani) for the positive assessment of our work and the crucial, actionable feedback regarding data availability.

General Comment:

“The study addresses an important educational gap... The methodology and interpretation are appropriate, and the manuscript is generally clear and coherent.”

Author Response: We are grateful for the reviewer’s positive and encouraging assessment of our work’s significance and execution.

Specific Major Comment:

“Data Availability Concern: The statement ‘data available from the corresponding author upon request’ does not comply with the PLOS ONE Data Policy... Authors should deposit all underlying data... in a public repository (e.g., Figshare, Dryad) and update the Data Availability Statement...”

Author Response: We fully agree with the reviewer on the importance of data transparency and reproducibility. As detailed in our response to the editor’s point #2 above, we have acted upon this essential recommendation. We have deposited the complete dataset in Figshare, a recommended public repository, and have updated the Data Availability Statement in the manuscript to provide the direct, permanent DOI link. This ensures full, unrestricted access to the data, significantly enhancing the study’s transparency and compliance with journal standards.

Additional Checks:

“Is the manuscript presented in an intelligible fashion and written in standard English?”

Author Response: Following the reviewer’s confirmation that the manuscript is clear, we have nonetheless conducted a thorough proofreading of the revised text to correct any minor typographical or grammatical errors, further improving the clarity of the presentation.

Attachment Submitted filename: Response to Reviewers.docx

# Peer review: Decision Letter 1

**DOI:** 10.1371/journal.pone.0351886.r003

**Authors:** Yagnik Dave

28 Apr 2026

PONE-D-25-36678R1

Clinical Practice Teaching System for MNS Cardiac Rehabilitation: Delphi Consensus

PLOS One

Dear Dr. wang,

Thank you for submitting your manuscript to PLOS ONE. After careful consideration, we feel that it has merit but does not fully meet PLOS ONE’s publication criteria as it currently stands. Therefore, we invite you to submit a revised version of the manuscript that addresses the points raised during the review process.

==============================

**Before preceeding further authors must have to adress below mention concerned arise by the reviewer**

Tone down claims of “strong consensus” and revise to reflect moderate agreement (as per Kendall’s W values).

Improve English language quality (grammar, clarity, and reduce repetition in Discussion).

Clarify ethical statement—Delphi studies involve human participants; include IRB approval or exemption with justification and reference number.

Add a clear limitation stating that the framework lacks prospective clinical validation.

Consider moving lengthy Tables (3–7) to Supplementary Material to improve readability.

==============================

Please submit your revised manuscript by Jun 12 2026 11:59PM. If you will need more time than this to complete your revisions, please reply to this message or contact the journal office at plosone@plos.org. When you're ready to submit your revision, log on to https://www.editorialmanager.com/pone/ and select the 'Submissions Needing Revision' folder to locate your manuscript file.

Please include the following items when submitting your revised manuscript:

- A letter that responds to each point raised by the academic editor and reviewer(s). You should upload this letter as a separate file labeled 'Response to Reviewers'.
- A marked-up copy of your manuscript that highlights changes made to the original version. You should upload this as a separate file labeled 'Revised Manuscript with Track Changes'.
- An unmarked version of your revised paper without tracked changes. You should upload this as a separate file labeled 'Manuscript'.

If you would like to make changes to your financial disclosure, please include your updated statement in your cover letter. Guidelines for resubmitting your figure files are available below the reviewer comments at the end of this letter.

If applicable, we recommend that you deposit your laboratory protocols in protocols.io to enhance the reproducibility of your results. Protocols.io assigns your protocol its own identifier (DOI) so that it can be cited independently in the future. For instructions see: https://journals.plos.org/plosone/s/submission-guidelines#loc-laboratory-protocols. Additionally, PLOS ONE offers an option for publishing peer-reviewed Lab Protocol articles, which describe protocols hosted on protocols.io. Read more information on sharing protocols at https://plos.org/protocols?utm_medium=editorial-email&utm_source=authorletters&utm_campaign=protocols.

As the corresponding author, your ORCID iD is verified in the submission system and will appear in the published article. PLOS supports the use of ORCID, and we encourage all coauthors to register for an ORCID iD and use it as well. Please encourage your coauthors to verify their ORCID iD within the submission system before final acceptance, as unverified ORCID iDs will not appear in the published article. *Only*   the individual author can complete the verification step; PLOS staff *cannot*   verify ORCID iDs on behalf of authors.

We look forward to receiving your revised manuscript.

Kind regards,

Yagnik Dave

Academic Editor

PLOS One

**Journal Requirements:**

1. If the reviewer comments include a recommendation to cite specific previously published works, please review and evaluate these publications to determine whether they are relevant and should be cited. There is no requirement to cite these works unless the editor has indicated otherwise.

2. Please review your reference list to ensure that it is complete and correct. If you have cited papers that have been retracted, please include the rationale for doing so in the manuscript text, or remove these references and replace them with relevant current references. Any changes to the reference list should be mentioned in the rebuttal letter that accompanies your revised manuscript. If you need to cite a retracted article, indicate the article’s retracted status in the References list and also include a citation and full reference for the retraction notice.

[Note: HTML markup is below. Please do not edit.]

Reviewers' comments:

Reviewer's Responses to Questions

**Comments to the Author**

1. If the authors have adequately addressed your comments raised in a previous round of review and you feel that this manuscript is now acceptable for publication, you may indicate that here to bypass the “Comments to the Author” section, enter your conflict of interest statement in the “Confidential to Editor” section, and submit your "Accept" recommendation.

Reviewer #1: All comments have been addressed

**********

2. Is the manuscript technically sound, and do the data support the conclusions?

The manuscript must describe a technically sound piece of scientific research with data that supports the conclusions. Experiments must have been conducted rigorously, with appropriate controls, replication, and sample sizes. The conclusions must be drawn appropriately based on the data presented.

Reviewer #1: Yes

**********

3. Has the statistical analysis been performed appropriately and rigorously?

Reviewer #1: Yes

**********

4. Have the authors made all data underlying the findings in their manuscript fully available?

The PLOS Data policy requires authors to make all data underlying the findings described in their manuscript fully available without restriction, with rare exception (please refer to the Data Availability Statement in the manuscript PDF file). The data should be provided as part of the manuscript or its supporting information, or deposited to a public repository. For example, in addition to summary statistics, the data points behind means, medians and variance measures should be available. If there are restrictions on publicly sharing data—e.g. participant privacy or use of data from a third party—those must be specified.

Reviewer #1: Yes

**********

5. Is the manuscript presented in an intelligible fashion and written in standard English?

PLOS ONE does not copyedit accepted manuscripts, so the language in submitted articles must be clear, correct, and unambiguous. Any typographical or grammatical errors should be corrected at revision, so please note any specific errors here.

Reviewer #1: Yes

**********

6. Review Comments to the Author

Please use the space provided to explain your answers to the questions above. You may also include additional comments for the author, including concerns about dual publication, research ethics, or publication ethics. (Please upload your review as an attachment if it exceeds 20,000 characters)

Reviewer #1: Mandatory minor revisions before acceptance:

Tone down claims of “strong consensus” and revise to reflect moderate agreement (as per Kendall’s W values).

Improve English language quality (grammar, clarity, and reduce repetition in Discussion).

Clarify ethical statement—Delphi studies involve human participants; include IRB approval or exemption with justification and reference number.

Add a clear limitation stating that the framework lacks prospective clinical validation.

Consider moving lengthy Tables (3–7) to Supplementary Material to improve readability.

**********

7. PLOS authors have the option to publish the peer review history of their article (what does this mean?). If published, this will include your full peer review and any attached files.

If you choose “no”, your identity will remain anonymous but your review may still be made public.

**Do you want your identity to be public for this peer review?**   For information about this choice, including consent withdrawal, please see our Privacy Policy.

Reviewer #1: **Yes:**  Dr Hemangkumar Sudhakarbhai Jani

**********

[NOTE: If reviewer comments were submitted as an attachment file, they will be attached to this email and accessible via the submission site. Please log into your account, locate the manuscript record, and check for the action link "View Attachments". If this link does not appear, there are no attachment files.]

To ensure your figures meet our technical requirements, please review our figure guidelines: https://journals.plos.org/plosone/s/figures

You may also use PLOS’s free figure tool, NAAS, to help you prepare publication quality figures: https://journals.plos.org/plosone/s/figures#loc-tools-for-figure-preparation.

NAAS will assess whether your figures meet our technical requirements by comparing each figure against our figure specifications.

# Author comment: Author response to Decision Letter 2

**DOI:** 10.1371/journal.pone.0351886.r004

31 May 2026

To the Academic Editor, Dr. Yagnik Dave, and the Reviewer,

We thank you for the thoughtful and constructive comments on our manuscript. We have carefully addressed all the points raised by the academic editor and reviewers. Below we provide a point‑by‑point response. All changes in the manuscript have been highlighted in the “Revised Manuscript with Track Changes” file.

Journal Requirements (Editor’s Comments):

Comment 1

“Tone down claims of ‘strong consensus’ and revise to reflect moderate agreement (as per Kendall’s W values).”

Response: We agree with the reviewer. The Kendall’s W values ranged from 0.13 to 0.21, which indicates moderate, not strong, agreement among experts. Accordingly, we have revised the manuscript as follows:

Abstract, Results section:

Original: “The Delphi study demonstrated high engagement … with robust validity (Cr=0.83-0.84; Kendall's W=0.13-0.21, p<0.05). Consensus yielded a structured framework…”

Revised to: “The Delphi study demonstrated high engagement … and acceptable expert authority (Cr = 0.83‑0.84). Kendall’s W values ranged from 0.13 to 0.21 (p < 0.05), indicating moderate agreement among experts. The consensus process yielded a structured framework…”

Results, “Results of the First Delphi Survey”:

Original: “The second Delphi round confirmed unanimous expert consensus on all items…”

Revised to: “The second Delphi round confirmed consistent expert agreement on all items…”

No other statements implying “strong consensus” remain in the manuscript.

Comment 2

“Improve English language quality (grammar, clarity, and reduce repetition in Discussion).”

Response: We have thoroughly revised the Discussion section to improve grammar, clarity, and conciseness. Major changes include:

Corrected grammatical errors (e.g., “This study is kind to pinpoint” → “This study identified”).

Removed redundant content (e.g., duplicate lists of interventions such as nutrition counseling, weight management, etc.).

Simplified overly complex sentences and improved logical flow.

Clarified ambiguous pronoun references (e.g., “which encourages medical schools” → “Medical schools should integrate…”).

Reorganized the Discussion into clearer thematic paragraphs.

The revised Discussion is now more readable and free of repetition. All changes are visible in the “Revised Manuscript with Track Changes” file.

Comment 3

“Clarify ethical statement—Delphi studies involve human participants; include IRB approval or exemption with justification and reference number.”

Response: We thank the reviewer for this important correction. The original statement (“does not involve human experiments”) was inaccurate. We have revised the Ethical Considerations section as follows:

“This study was conducted in accordance with the Declaration of Helsinki (as revised in 2013). The research involved human participants (expert panel members) but did not include patients, clinical interventions, or any identifiable sensitive data. According to the policy of the Institutional Review Board of Binzhou Medical University, formal ethics approval is not required for studies that solely collect anonymous expert opinions through surveys without intervention or patient contact. Therefore, this study was exempt from full ethical review. Nevertheless, all participating experts provided electronic informed consent prior to completing the questionnaire. They were fully informed of the study purpose, the voluntary nature of participation, and their right to withdraw at any time without negative consequences. Anonymity and confidentiality were strictly maintained throughout the study. (No reference number was issued under this exemption; written confirmation from the IRB can be provided upon request.)”

Thus, we now explicitly acknowledge human participation, provide the exemption justification based on institutional policy, and note the absence of a reference number (while offering to provide IRB confirmation if needed).

Comment 4

“Add a clear limitation stating that the framework lacks prospective clinical validation.”

Response: We agree and have added this limitation. In the Limitations section, we now state:

“Second, the developed teaching framework lacks prospective clinical validation. Although the Delphi method achieved moderate expert consensus, the framework’s effectiveness, feasibility, and impact on student learning outcomes have not yet been tested in real‑world clinical teaching settings.”

We also revised the future research direction accordingly: “Future research should focus on the prospective clinical application of this system using rigorous study designs (e.g., pre‑post comparisons or cluster randomized trials).”

The full revised Limitations paragraph is included in the track‑changed manuscript.

Comment 5

“Consider moving lengthy Tables (3–7) to Supplementary Material to improve readability.”

Response: We agree with the reviewer. To improve the readability of the main manuscript, we have:

Removed the full Tables 3, 4, 5, 6, and 7 from the Results section.

Added a sentence in the “Results of the First Delphi Survey” paragraph: “The detailed indicator lists with importance ratings, coefficients of variation, and weights are provided in Supplementary Tables S1–S5 (see Supplementary File 1).”

Created a separate Supplementary Material file containing Supplementary Tables S1–S5 (which correspond to original Tables 3–7).

Retained Tables 1 and 2 (search strategy and participant demographics) in the main text, as they are short and essential for understanding.

This change significantly improves the flow of the manuscript while preserving all detailed data for interested readers.

We believe the revised manuscript is substantially improved and hope it now meets the journal’s standards for publication. We appreciate your time and expertise in reviewing this work.

Sincerely,

*Yanyan Fan

Binzhou Medical University

Addr: Binzhou Medical University, No. 346 Guanhai Road, Binhai Road Street, Yantai City, Shandong Province, China

E-mail: fanyanyan@bzmc.edu.cn

*Guiyun Wang

Shandong Xiehe University

Addr: Shandong Xiehe University, No. 6277, Jiqing Road, Licheng District, Jinan City, Shandong Province, China

E-mail: wangguiyun@sdxiehe.edu.cn

Attachment Submitted filename: Response_to_Reviewers_auresp_2.docx

# Editor report: Decision Letter 2

**DOI:** 10.1371/journal.pone.0351886.r005

**Authors:** Yagnik Dave

2 Jun 2026

<p>Clinical Practice Teaching System for MNS Cardiac Rehabilitation: Delphi Consensus

PONE-D-25-36678R2

Dear Dr.  guiyun wan

We’re pleased to inform you that your manuscript has been judged scientifically suitable for publication and will be formally accepted for publication once it meets all outstanding technical requirements.

Within one week, you’ll receive an e-mail detailing the required amendments. When these have been addressed, you’ll receive a formal acceptance letter and your manuscript will be scheduled for publication.

An invoice will be generated when your article is formally accepted. Please note, if your institution has a publishing partnership with PLOS and your article meets the relevant criteria, all or part of your publication costs will be covered. Please make sure your user information is up-to-date by logging into Editorial Manager at Editorial Manager® and clicking the ‘Update My Information' link at the top of the page. For questions related to billing, please contact billing support.

If your institution or institutions have a press office, please notify them about your upcoming paper to help maximize its impact. If they’ll be preparing press materials, please inform our press team as soon as possible -- no later than 48 hours after receiving the formal acceptance. Your manuscript will remain under strict press embargo until 2 pm Eastern Time on the date of publication. For more information, please contact onepress@plos.org.

Kind regards,

Yagnik Dave

Academic Editor

PLOS One

Additional Editor Comments (optional):

Reviewers' comments:

# Editor report: Acceptance letter

**DOI:** 10.1371/journal.pone.0351886.r006

**Authors:** Yagnik Dave

PONE-D-25-36678R2

PLOS One

Dear Dr. wang,

I'm pleased to inform you that your manuscript has been deemed suitable for publication in PLOS One. Congratulations! Your manuscript is now being handed over to our production team.

At this stage, our production department will prepare your paper for publication. This includes ensuring the following:

* All references, tables, and figures are properly cited

* All relevant supporting information is included in the manuscript submission,

* There are no issues that prevent the paper from being properly typeset

You will receive further instructions from the production team, including instructions on how to review your proof when it is ready. Please keep in mind that we are working through a large volume of accepted articles, so please give us a few days to review your paper and let you know the next and final steps.

Lastly, if your institution or institutions have a press office, please let them know about your upcoming paper now to help maximize its impact. If they'll be preparing press materials, please inform our press team within the next 48 hours. Your manuscript will remain under strict press embargo until 2 pm Eastern Time on the date of publication. For more information, please contact onepress@plos.org.

You will receive an invoice from PLOS for your publication fee after your manuscript has reached the completed accept phase. If you receive an email requesting payment before acceptance or for any other service, this may be a phishing scheme. Learn how to identify phishing emails and protect your accounts at https://explore.plos.org/phishing.

If we can help with anything else, please email us at customercare@plos.org.

Thank you for submitting your work to PLOS ONE and supporting open access.

Kind regards,

PLOS ONE Editorial Office Staff

on behalf of

Dr. Yagnik Dave

Academic Editor

PLOS One
